# Role of Ultrasound-Guided Marker Placement in Breast Lesion for Precise Localization, Surgical Resection, Neoadjuvant Chemotherapy Response, and Post-Chemotherapy Follow-Up

**DOI:** 10.7759/cureus.89402

**Published:** 2025-08-05

**Authors:** Suneela Shaukat, Javeria Haroon, Faiza Afzal, Sobia Mazhar, Zara Shaukat, Zonia Ghaffar, Mah Jabeen Masood

**Affiliations:** 1 Department of Diagnostic Radiology, King Edward Medical University, Lahore, PAK; 2 Department of Diagnostic Radiology, Shaukat Khanum Memorial Cancer Hospital and Research Centre, Lahore, PAK; 3 Department of Diagnostic Radiology, Sandwell and West Birmingham National Health Service (NHS) Trust, Birmingham, GBR

**Keywords:** breast conservation surgery, breast lesions, interventional radiology, neo-adjuvant chemotherapy, ultrasound-guided markers

## Abstract

Objective

To evaluate the effectiveness of ultrasound-guided marker placement in the precise localization of breast lesions, facilitate accurate surgical resection, and monitor tumor response during and after neoadjuvant chemotherapy.

Materials and methods

From January 2022 to December 2022, 70 female patients with breast carcinoma underwent a trial of metallic marker insertion into the tumor. The markers were made by cutting 5CC disposable syringe needles having a total length of 5 mm. These markers were inserted using a 16 G LP needle having a length of 10 cm.

Results

Among 70 female breast cancer patients (mean age 47.9 ± 8.0 years), ultrasound-guided metallic markers enabled precise tumor localization before, during, and after neoadjuvant chemotherapy. In 45 patients receiving chemotherapy, tumor size significantly decreased from 3.66 ± 0.69 cm to 0.61 ± 0.21 cm (p < 0.00001). Marker-guided surgery achieved complete tumor excision with negative margins. Intraoperative imaging and postoperative histopathology confirmed accurate localization and successful resection.

Conclusion

Ultrasound-guided marker placement enables precise tumor localization after neoadjuvant chemotherapy, improving surgical accuracy and breast cancer treatment outcomes.

## Introduction

Breast cancer is a malignant tumor that originates in the cells of the breast [1]. It remains the leading cause of cancer-related deaths among women worldwide. Although incidence rates vary significantly across different regions, a global increase in cases has been observed, including in areas that previously reported low prevalence [2].

Several well-established risk factors are linked to breast cancer, many of which involve estrogen exposure. Early onset of menstruation, late onset of menopause, and postmenopausal obesity are among the factors that can elevate the risk [3]. However, it is important to recognize that not all women with these risk factors will develop breast cancer, and some may develop the disease without any known risk factors [4]. Therefore, early detection through screening tools such as mammography is vital for identifying breast cancer in its initial, more treatable stages.

Recent advancements in imaging, therapeutic techniques, and monitoring methods have significantly improved early detection and diagnosis of both primary and secondary malignancies [5]. Despite these developments, small or deeply located tumors remain challenging to treat. 

In certain clinical scenarios, lesion marking is essential to enable accurate targeting, avoiding incomplete removal or unnecessary excision of healthy tissue [6]. To address this, Kanazawa et al. introduced a hook-wire/suture system in 1993 to assist in localizing lung lesions during thoracoscopy. Since then, various targeting systems have been developed, highlighting the increasing role of interventional radiology (IR) procedures in cancer management [7].

For non-palpable breast lesions identifiable only through imaging, surgeons often rely on image-guided localization techniques [8]. These methods help accurately locate and remove the lesion while preserving healthy tissue, improving cosmetic outcomes. The primary goal is to achieve an R0 resection, meaning complete tumor removal with negative margins, which is critical to minimize the risk of recurrence [9,10].

As image-guided biopsy has become standard practice, the placement of tissue markers at the biopsy site has become an integral part of the procedure [11,12]. Initially, tissue markers were used only when the target became indistinguishable after biopsy [13]. However, their routine use today is largely due to the improved ability to accurately sample smaller abnormalities, along with the use of larger-gauge biopsy devices that can sometimes distort or completely remove the visible portion of the target lesion.

## Materials and methods

This prospective descriptive cross-sectional study aimed to evaluate the effectiveness of ultrasound-guided metallic marker placement in (1) precise localization of breast tumors before and after neoadjuvant chemotherapy, (2) monitoring tumor size reduction during treatment, and (3) confirming complete tumor excision during surgery. The study was conducted at the Radiology Department of Mayo Hospital, Lahore, in collaboration with the Oncology and General Surgery departments, from January 2022 to December 2022, following Institutional Review Board (IRB) approval. Patients were eligible for inclusion if they were female, aged between 35 and 68 years, and had a histologically confirmed diagnosis of invasive breast carcinoma. Only those with non-metastatic disease (clinical stages I-III), as determined by clinical and radiological evaluation, were included. Eligible patients were scheduled to undergo neoadjuvant chemotherapy and/or surgical resection as part of their treatment plan and had ultrasound-visible breast lesions amenable to marker placement. All participants provided written informed consent prior to enrollment in the study. Exclusion criteria included those patients who had non-palpable, ultrasound-invisible tumors not suitable for ultrasound-guided marker placement; had received prior chemotherapy, radiotherapy, or surgery for the breast cancer; were diagnosed with inflammatory breast cancer or had metastatic disease (Stage IV) at presentation; were pregnant or lactating; had a known allergy or contraindication to marker material (e.g., stainless steel); and were unable or unwilling to comply with study procedures or follow-up. We observed cultural ethics. The information was kept confidential.

The study included 70 patients who met the selection criteria. The informed consent was taken. A questionnaire was prepared for data collection. The affected breast tissue was examined using a high-frequency ultrasound transducer. Ultrasonography was performed before the implantation of markers, during chemotherapy, and one week before breast surgery. The exact distance from the mass to the nipple was measured using ultrasonography. Ultrasound was performed during chemotherapy cycles to track interim changes in tumor size and marker position, facilitating early assessment of treatment response.

**Figure 1:**
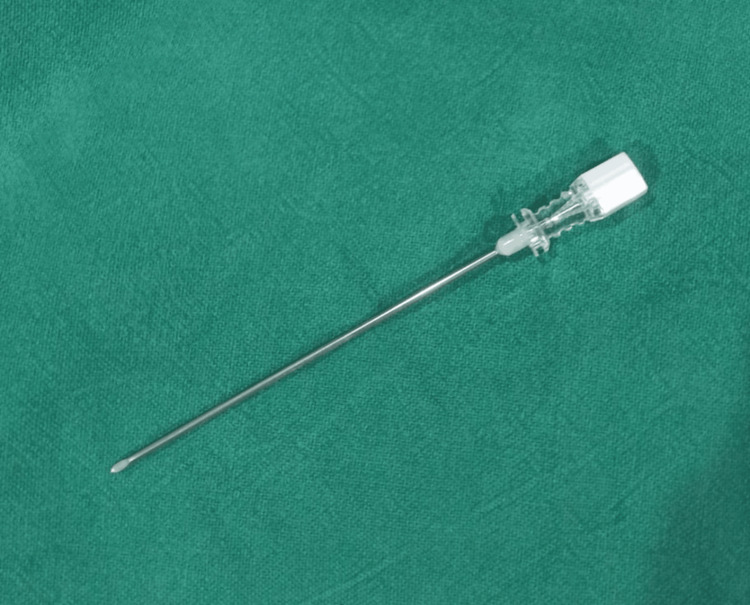
Showing a 16G LP needle for insertion of markers in breast tissue

**Figure 2:**
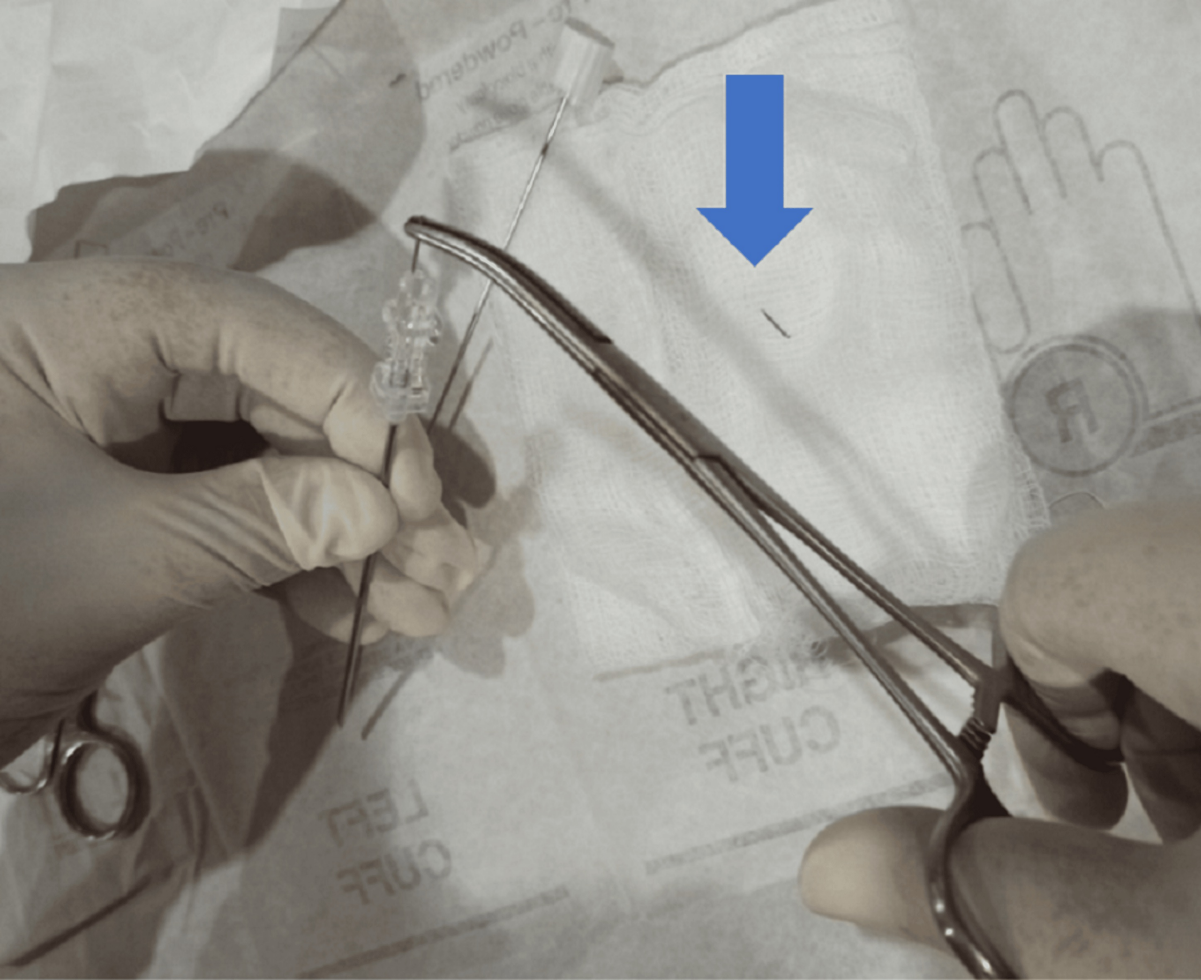
Arrow showing small metallic markers made from cutting 5 CC disposable syringe needles

Markers were typically made of stainless steel by cutting the needles of a 5 cc disposable syringe with a total length of 5 mm. These markers were inserted by using 16 G and 10 cm long needles (Figures 1, 2).

First, the area was cleaned using povidone iodine, the skin was infiltrated using lignocaine, a 16-gauge needle was inserted, and the markers were seen passing out from the bevel. Two or three markers were placed in all masses, depending on their size. If the size was small, one marker was placed at the center. In larger lesions, one marker was placed in the center, and others were placed at the periphery of the lesion. The markers were inserted by the same radiologist who had previously performed ultrasonography. All 70 patients underwent marker insertion. Of these 70 patients, 45 women with breast cancer underwent neoadjuvant chemotherapy followed by surgery, and only surgical resection was performed in the remaining 25 patients. The patients were referred for post-neoadjuvant chemotherapy and post-surgical follow-up.

IBM Corp. Released 2019. IBM SPSS Statistics for Windows, Version 28.0. Armonk, NY: IBM Corp. was used to enter and analyze the data. All data were presented as mean ± standard deviation with an alpha of 0.05. We used the same information sources and questionnaire in this study to prevent bias. All ultrasound procedures were performed using the Esaote MyLab™ (Genoa, Italy). The ultrasound system is equipped with a high-frequency linear transducer operating at 8-10 MHz. The examinations and marker placements were conducted and interpreted by two experienced radiologists, each with five years of experience in breast imaging, to ensure consistency and accuracy of evaluation. Intraoperative radiography and postoperative histopathology of the resected tumors were performed to confirm the R0 resection status.

## Results

The study included 70 (100%) female patients diagnosed with breast carcinoma. The mean age of participants was 47.9 ± 8.0 years (range: 35-68 years). A total of 62 patients (88.57%) presented with a single lesion, whereas eight (11.42%) had additional satellite lesions. Histologically, invasive ductal carcinoma was the most common subtype, accounting for 55 (78.57%) cases, followed by invasive lobular carcinoma in 10 (14.28%) and a mixed pattern of both types in five (7.14%) patients (Table 1). Ultrasound-guided metallic markers were placed in all participants, typically 1-3 markers depending on the size of the tumor, with positions confirmed by ultrasound and mammography. These markers enabled accurate tumor localization at multiple stages: before treatment, during chemotherapy, preoperatively, and in follow-up. Preoperative measurements from the nipple and skin surface to the marker were recorded to aid intraoperative localization. Surgeons used these distances for surface marking to guide excision, and in some cases, markers were also palpated during surgery. This technique proved especially useful for small, deep, or peripherally located tumors and in patients with large or pendulous breasts. While postoperative imaging confirmed the absence of markers in resected areas, this was not used as a surrogate for complete tumor excision. Confirmation of R0 resection status was based on post-surgical histopathology, which remains the definitive standard for assessing margin status.

**Table 1:** Initial details of patients

Characteristics	Parameter	Numbers (n=70)	Percentage
Gender	Females	70	100%
Lesions	Single lesion	62	88.57%
	Satellite lesion	08	11.42%
Carcinoma type	Invasive Ductal	55	78.57%
	Invasive lobular	10	14.28%
	Both Ductal & lobular	05	07.14%

**Figure 3:**
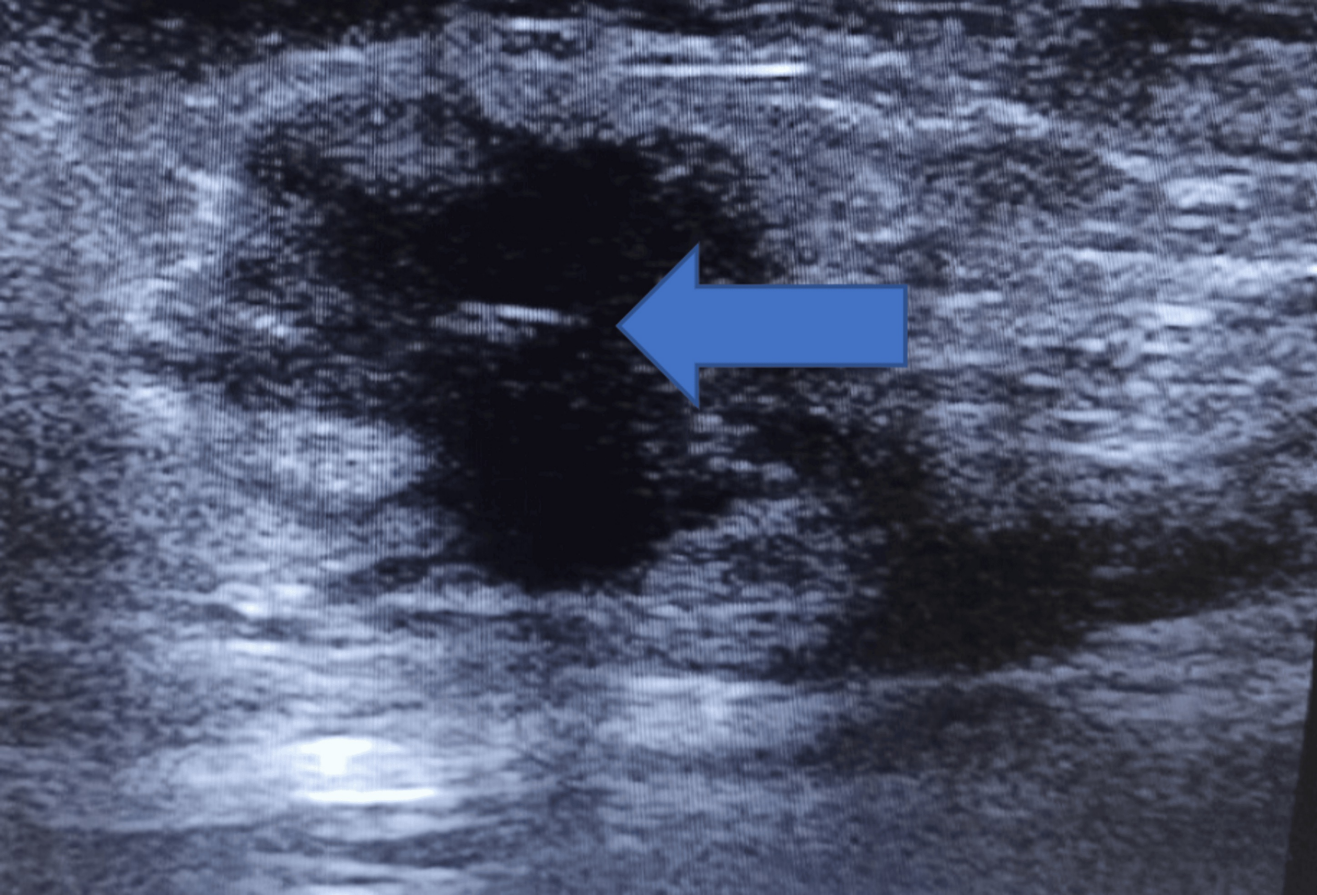
Pre-chemotherapy image showing marker in center of lesion

**Figure 4:**
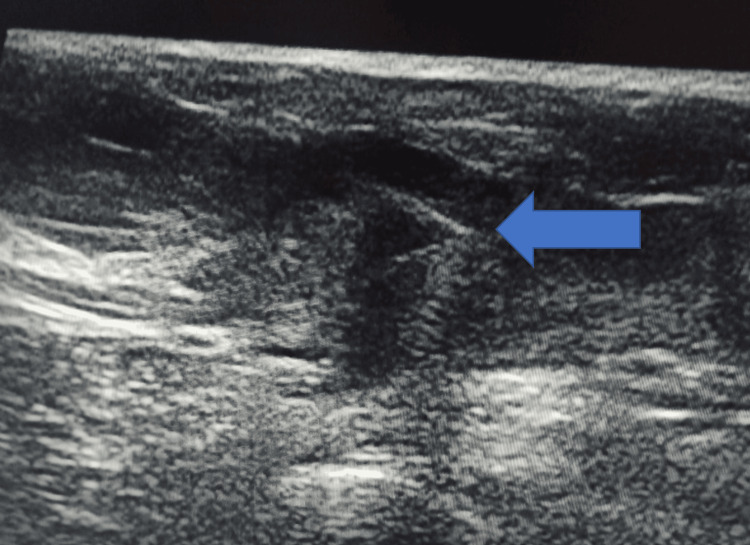
The post-chemotherapy image shows the reduction in size of the tumor that became impalpable clinically, and the marker led to surface marking of the lesion under ultrasound guidance, and the surgeons did the lumpectomy of the patient

Among the 70 patients, 45 (64.2%) underwent neoadjuvant chemotherapy; the mean tumor size was 3.66 ± 0.69 cm before chemotherapy, 1.43 ± 0.37 cm during chemotherapy, and 0.61 ± 0.21 cm after chemotherapy. Paired t-tests confirmed statistically significant reductions in tumor size at each treatment stage (p < 0.00001 for all comparisons) (Figures 3, 4).

**Figure 5:**
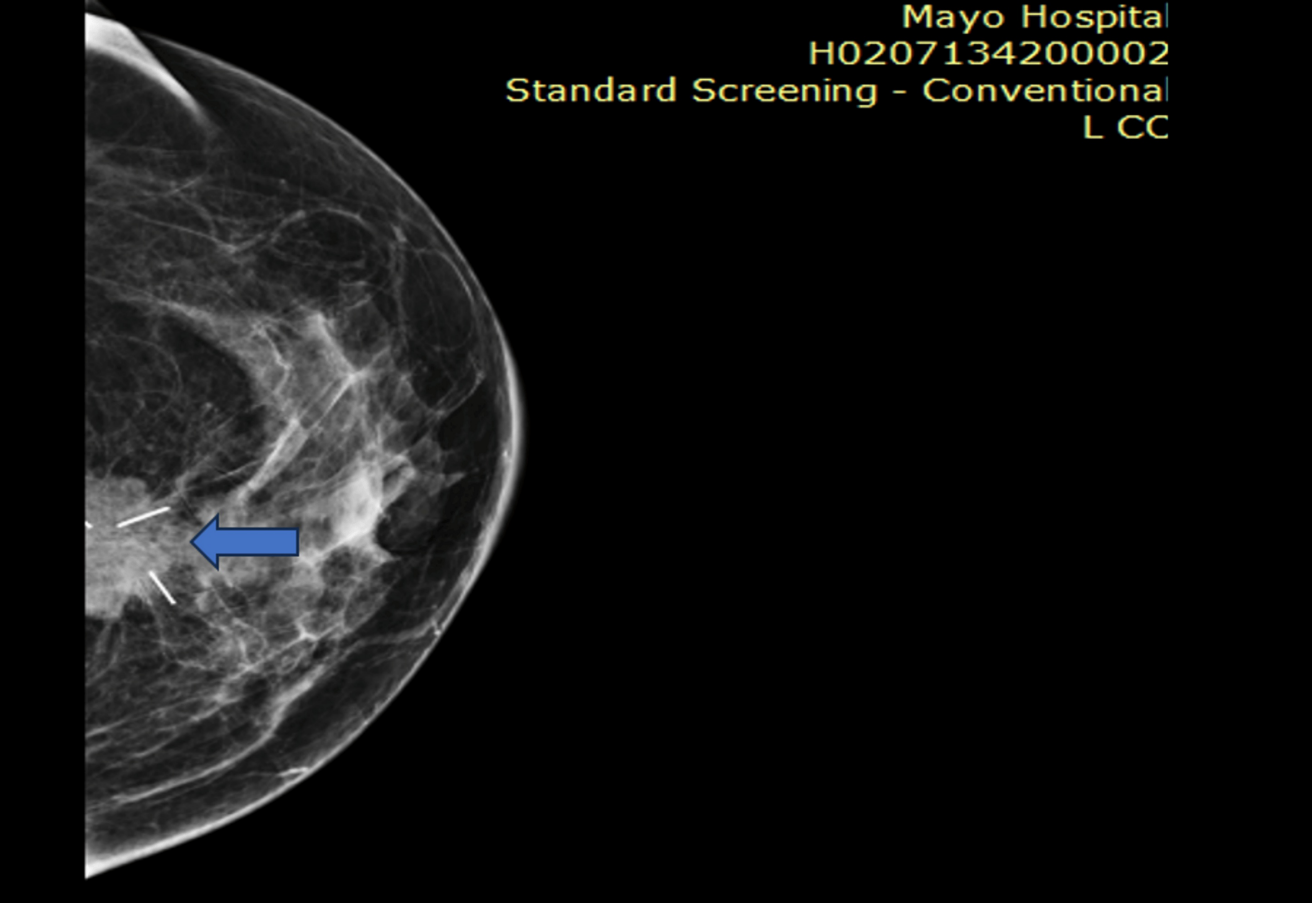
In a pre-chemotherapy mammogram, an arrow shows metallic markers in a breast lesion

**Figure 6:**
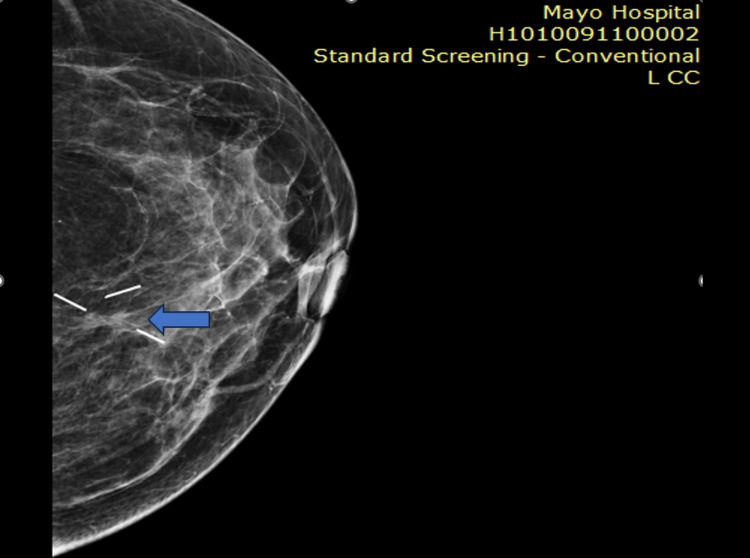
In the post-chemotherapy mammogram, the mass disappeared, but metallic clips can be seen

Among those 45 (64.2%) patients treated with chemotherapy, tumor size progressively decreased from an initial range of 0.8 to 4.5 cm at presentation to 0.4 to 3.5 cm at the middle of chemotherapy cycles, and with completion of chemotherapy finally to 0.2 to 0.8 cm in the pre-operative period, demonstrating a significant treatment response (Table 2) (Figures 5, 6).

**Table 2:** Demonstrates shrinkage of the tumor size after chemotherapy (n=45)

Time of measurement	Size of tumor (mean ± SD)
At presentation	3.66 ± 0.69 cm
At follow-up during chemotherapy	1.43 ± 0.37 cm
Pre-operative (completion of chemotherapy)	0.61 ± 0.21 cm

**Figure 7:**
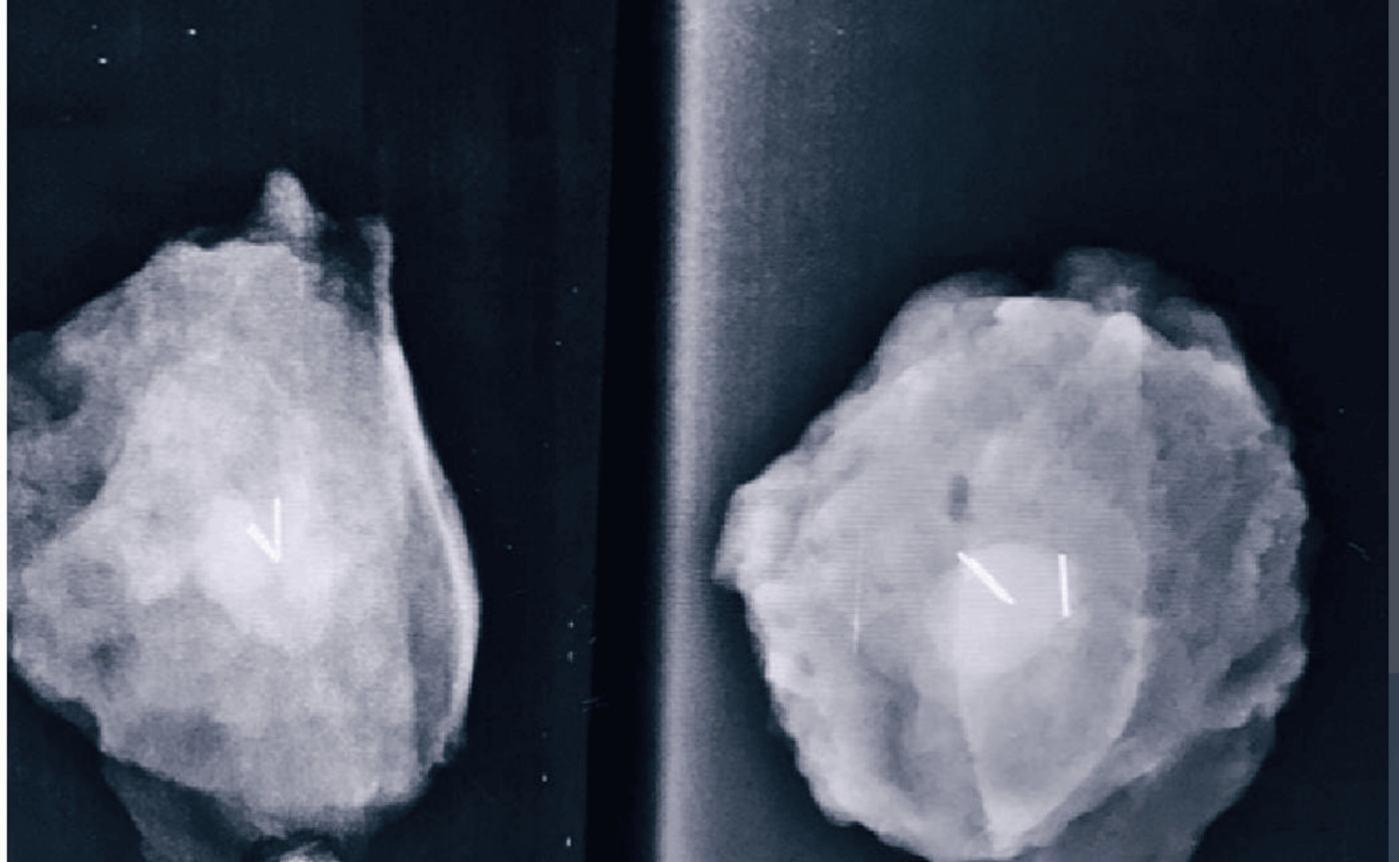
Intraoperative radiograph of the resected specimen demonstrating complete excision of a clinically non-palpable breast lesion, with surgical clips visible at the resection site

All 45 patients who received neoadjuvant chemotherapy subsequently underwent surgical resection. Markers on intraoperative imaging of resected specimens supported the success of the localization and resection of tumors. Post-operative imaging confirmed complete tumor excision, with all patients achieving tumor-free (negative) surgical margins on histopathology (Figure 7).

In a subgroup of 25 (35.7%) patients who underwent surgical resection of a mass, preoperative ultrasound identified tumor markers with tumor sizes ranging from 1.4 to 4.5 cm. Postoperative imaging and histopathology confirmed the absence of these markers, indicating complete tumor excision (Table 3).

**Table 3:** Demonstrates tumor size before and after surgical resection of breast lesion (n=25)

Time at measurement	Ultrasound findings	Tumor size
Pre-operative	Markers identified	4.26 ± 0.78 cm
Post-Operative	No Markers	N/A

The use of ultrasound-guided metallic markers greatly facilitated tumor localization, supporting both neoadjuvant therapy planning and surgical intervention. Additionally, changes in marker position and tumor size effectively reflected tumor regression following chemotherapy.

## Discussion

Advancements in imaging technologies for non-palpable breast lesions have led to the widespread adoption of image-guided biopsy and localization techniques [14]. These minimally invasive breast core biopsy methods now serve as a reliable diagnostic approach and have largely supplanted surgical excision as the preferred initial step in tissue evaluation [15].

Breast-conserving surgery involves complete excision of the tumor with clear margins, removal of ipsilateral lymph nodes, and subsequent radiation therapy to the affected breast [16]. This surgical approach aims not only to ensure effective local control of malignancy but also to preserve the aesthetic and functional integrity of the breast. The introduction of neoadjuvant cytotoxic chemotherapy and tamoxifen citrate therapy has further expanded the indications for breast-conserving surgery. Patients with initially inoperable large breast masses can now undergo this procedure following a favorable response to neoadjuvant therapy. Tumor shrinkage under these regimens enhances the possibility of complete resection while maintaining breast appearance and function. These therapeutic advancements have significantly improved both clinical outcomes and quality of life for individuals with breast cancer [17].

The use of blunt metal markers for localizing tumors in soft tissue has been practiced for over three decades in gynecological and head and neck cancers, with no documented marker migration [18]. Similarly, our study showed consistent results in breast cancer patients, with no observed displacement of the metallic markers relative to each other or to the original tumor location.

Ultrasound has proven to be an accurate modality for evaluating the size of breast tumors [14]. In line with existing literature and our findings, ultrasound reliably measured tumor response during chemotherapy, especially as lesions decreased in size [19,20].

Technological improvements have enhanced the detection of tissue markers on ultrasound. This includes measuring the distance between the tumor and the nipple, increasing the minimum number of markers used, and placing markers in clustered configurations [21]. Recording the tumor’s position relative to the nipple and its location at initial presentation proved valuable for accurate identification during follow-up assessments [22]. This was particularly beneficial for small or deeply located tumors, lesions situated at the periphery of the breast, and patients with large or drooping breasts [23]. The ultrasound-guided placement of metallic markers in or near the tumor has been shown to be especially effective in patients who experienced a complete pathological response after neoadjuvant chemotherapy. It addresses the clinical challenge of localizing tumors that are no longer detectable following successful treatment, a growing concern with the increasing use of breast-conserving surgery in this context [24]. While chemotherapy acts systemically and is not directed by anatomical localization, ultrasound-guided marker placement remains valuable for tracking tumor response and planning surgery. In our study, markers were used to document tumor location before chemotherapy and to assist in surgical excision after treatment, particularly in cases of complete or near-complete clinical response. Although our study did not directly measure whether marker placement improved R0 resection rates, all patients achieved tumor-free margins confirmed by histopathology, and marker-guided surface localization was used in all surgical cases.

Additionally, tissue marker placement plays a critical role in evaluating disease extent when multiple areas are biopsied. It facilitates correlation between imaging modalities, avoids redundant biopsies of benign areas, and ensures accurate tumor localization following neoadjuvant therapy [25]. Due to their high visibility on specimen radiographs, these markers also allow for immediate intraoperative confirmation of target lesion excision and help pathologists identify the precise area of interest in mastectomy specimens.

In our study, the small sample size, the inclusion of only female patients, and the single-center design may limit the generalizability of the findings. Additionally, the absence of a control group limits the ability to draw conclusions about the procedure’s clinical efficacy. The findings are primarily descriptive and observational, highlighting the feasibility and practical application of marker placement for tumor localization and tracking.

## Conclusions

Ultrasound-guided marker placement is a practical and cost-effective technique for localizing breast tumors in patients undergoing neoadjuvant chemotherapy followed by breast conservation surgery. In our cohort, metallic markers remained detectable throughout treatment and enabled consistent preoperative localization of tumors, including those that became clinically or radiologically occult. While all surgical cases achieved tumor-free margins on histopathology, further studies with control groups are needed to evaluate the direct impact of marker placement on surgical outcomes and long-term prognosis.
